# Identification and Evaluation of Cytotoxicity of Peptide Liposome Incorporated Citron Extracts in an in Vitro System

**DOI:** 10.3390/ijms19020626

**Published:** 2018-02-22

**Authors:** Xiaowei Zhang, Hee Jeong Yoon, Min Gyeong Kang, Gyeong Jin Kim, Sun Young Shin, Sang Hong Baek, Jung Gyu Lee, Jingjing Bai, Sang Yoon Lee, Mi Jung Choi, Kwonho Hong, Hojae Bae

**Affiliations:** 1 Department of Bioindustrial Technologies, College of Animal Bioscience and Technology, Konkuk University, Seoul 05029, Korea; zhangxiaowei9304@gmail.com (X.Z.); rose_0013@naver.com (H.J.Y.); vq1004pv@naver.com (M.G.K.); kkg0118@gmail.com (G.J.K.); 2 Laboratory of Cardiovascular Regeneration, Division of Cardiology, Seoul St. Mary’s Hospital, The Catholic University of Korea School of Medicine, Seoul 02841, Korea; tempast606@naver.com (S.Y.S.); whitesh@catholic.ac.kr (S.H.B.); 3 Department of Food Science and Biotechnology of Animal Resources, Sanghuh College of Life Sciences, Konkuk University, Seoul 02841, Korea; nocturne933@gmail.com (J.G.L.); baiijing@naver.com (J.B.); dhgus4211@naver.com (S.Y.L.); choimj@konkuk.ac.kr (M.J.C.); 4 Department of Stem Cell and Regenerative Biotechnology, KU Convergence Science and Technology Institute, Konkuk University, Seoul 05029, Korea; hongkh27@gmail.com

**Keywords:** citrus fruits, citron, nanotechnology, liposome, peptide, cell viability, cytotoxicity

## Abstract

Citrons have been widely used for medicinal purposes for a long time, but the application of citron in the food industry is still restricted. The extensive advantages of nanotechnology in the food industry have greatly broadened the application of foods. In this study, by employing nanotechnology, we prepared citron-extract nanoparticle with an average size of 174.11 ± 3.89 nm, containing protein peptide and/or liposome. In order to evaluate the toxicity of nanoparticles and to ensure food safety, biological cytotoxicity at the cell and genomic levels was also identified to examine the toxicity of citron extracts by using an in vitro system. Our results demonstrated that the cytotoxicity of citron_liposome_ was dependent on cell type in high concentrations (1 and 5 mg/mL), selectively against primary human cardiac progenitor cells (hCPCs), and human endothelial progenitor cells (hEPCs) in MTT and lactate dehydrogenase (LDH) assays. Interestingly, for the NIH-3T3 and H9C2 cell lines, cell cytotoxicity was observed with slight genotoxicity, especially from citron_peptide_ extract for both cell lines. Taken together, our study provides cytotoxicity data on nanoengineered citron extracts according to different cell type as is crucial for further applications.

## 1. Introduction

Citrus fruits have been consumed by humans for thousands of years, and are increasingly consumed worldwide for their nutritional and medicinal value [1]. Functional compounds, such as β-cryptoxanthin, β-carotene, folate, vitamin C, and quercetin are some recognized bioactive ingredients of citrus that defend against cancers [2,3,4,5]. For instance, some studies have reported that intake of citrus fruits results in reduced risk of breast and stomach cancers [6,7]. Other studies have focused on evaluating the antioxidant capacity of citrus extracts, their protective effect against oxidative damage, and other defensive responses against certain diseases such as lowering high blood-pressure risk or preventing kidney-stone formation [8,9]. However, despite the increased demand for citrus, studies related to the potential toxicity of citrus fruits are limited and restricted. Therefore, the focal point of our study was to investigate and identify the toxicity of citrus fruits to ensure their safety for various applications.

The citron (*Citrus medica*) is one of the three original citrus species and has already developed extensive applications for medicinal purposes, such as to treat sea sickness, pulmonary troubles, and intestinal ailments. With the improvement of processing technology, various citron fruit applications that maximize its functional advantages have been developed, but are still limited because of the botanical characteristics of the citron. Unlike other citrus fruits, its thick white rind is the main content of the citron and its pulp is dry with little juice [10]. Hence, citron has had only restricted use in the food industry, mainly as candied peel. Meanwhile, few studies have provided a biological examination of citron, such as of its bioactivity, pharmacology, toxicology, or bioavailability. Therefore, experimental studies to improve use of the citron and to determine its biological properties are essential.

Nanotechnology, as a cutting-edge scientific field, has many prospects for applications in various fields, such as medicine delivery, computer electronics, materials science, and for the food industry [11]. The application of nanotechnology in food science not only significantly increases the economic benefits, but also remarkably reforms food quality and safety [12,13,14]. Given the special nature of nanoparticles, the changes in the properties of food products are apparent, such as the improvement of food bioavailability, solubility, stability, biological activity, and safety [14]. To broaden the application of the citron and to further elaborate its biological benefits, the citron was prepared in nanometer scale by means of nanotechnology, and experiments were performed to measure its cell viability and genotoxicity by employing diverse in vitro models. By conducting comparative studies, we evaluated the different effects of citron extracts on different cell lines at the cellular and genomic levels.

## 2. Results

### 2.1. Measurement of Nanoparticles

**Figure 1:**
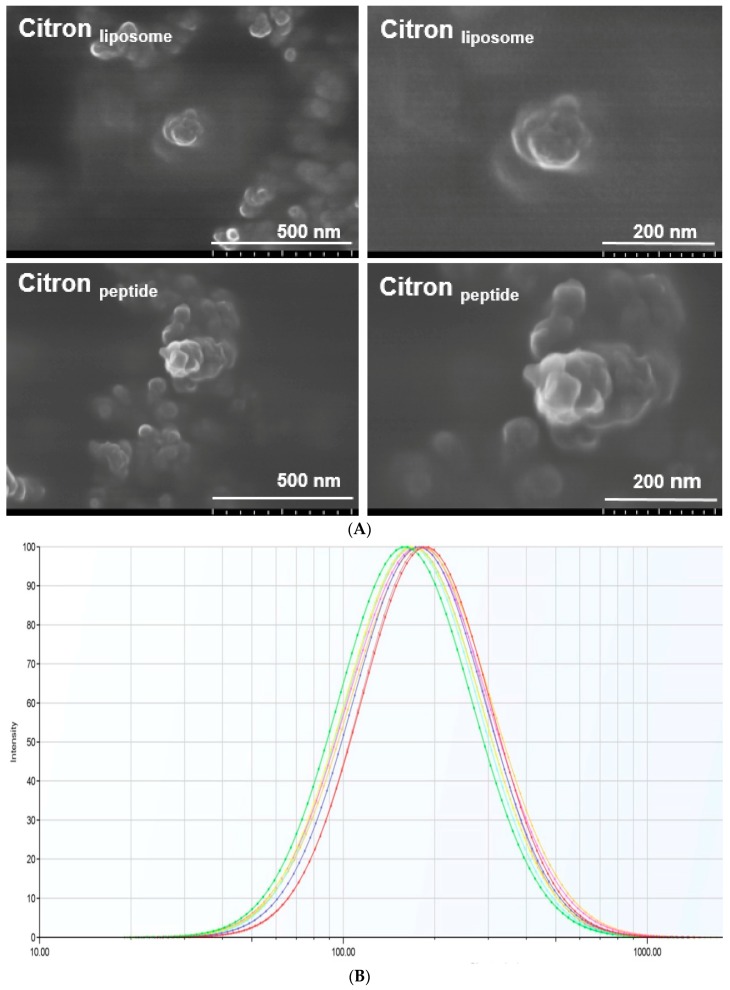
Nanoparticle measurements of citron, citron_peptide_, and citron_liposome_. (**A**) Citron extract powder was dispersed in PBS (10 mg/mL). Then 25–50 μL of dispersion was applied to a carbon tape and kept at room temperature until completely dried. After the gold coating, the morphology of the nanoparticles was analyzed by using field-emission scanning electron microscopy (FE-SEM, Hitachi SU-8010); (**B**) Citron_liposome_ was dispersed in PBS at a final concentration of 10 mg/mL. Then the size of samples was measured at 640 nm by NanoBrook Omni Analyzer. The curves showed similar output from three identical samples.

To evaluate the morphology and the size of citron nanoparticles, citron extracts were examined by using SEM and a Nano Size Analyzer (NanoBrook, Omni, Brookhaven Instruments Corporation, New York City, NY, USA). In our study, we labeled citron extract consisting of peptide with or without liposome as citron_liposome_ and citron_peptide_, respectively. The morphology of citron_peptide_ and citron_liposome_ is shown in Figure 1A. As can be seen from these images, typical particles within the size range of 120–150 nm were visible in both citron_peptide_ and citron_liposome_ test groups. In addition, Figure 1B shows the size distribution of citron extracts. Citron_peptide_ cannot be analyzed by the Nano Size Analyzer. However, in the case of citron_liposome_ group, nano-sized particles were detectable and exhibited a broad distribution with size, ranging from 60 to 300 nm (Figure 1B). The average size of citron_liposome_ nanoparticle was 174.11 ± 3.89 nm. 

### 2.2. Measurement of Cell Viability

In this study, three different types of citron extract samples (citron only, citron_peptide_, and citron_liposome_) were fabricated by using fish-skin peptide with or without liposome, as described in “Materials and Methods”. Liposome, with its typical physical and chemical properties, has had broad applications, ranging from the delivery of functional flavors and nutrients, food additives, and food antimicrobials [15,16]. Protein peptide, as materials for food encapsulation, also plays an important role in various food applications and therefore we utilized nanoengineered liposome and peptide to extend citron usage in food products, and evaluate the potential biological cytotoxicity [17]. 

**Figure 2:**
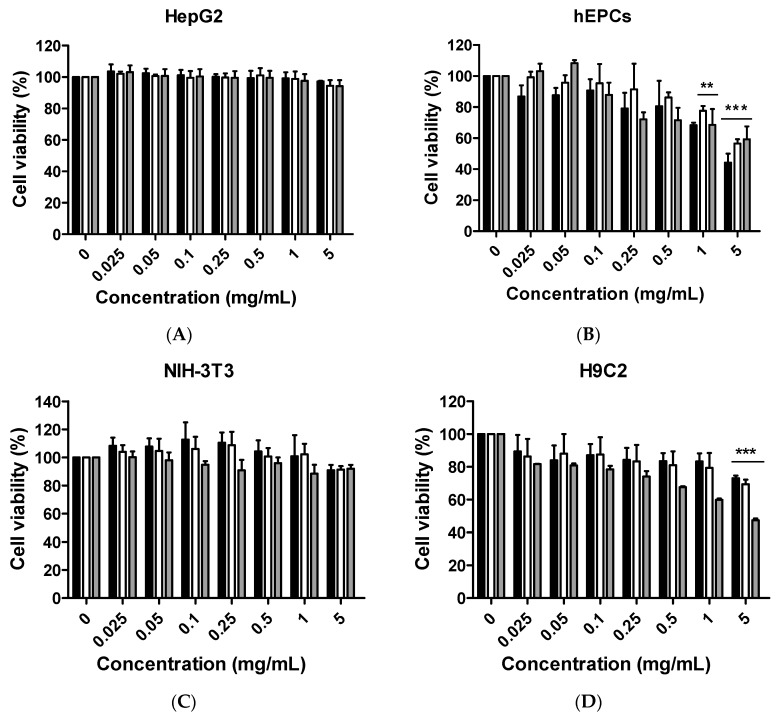

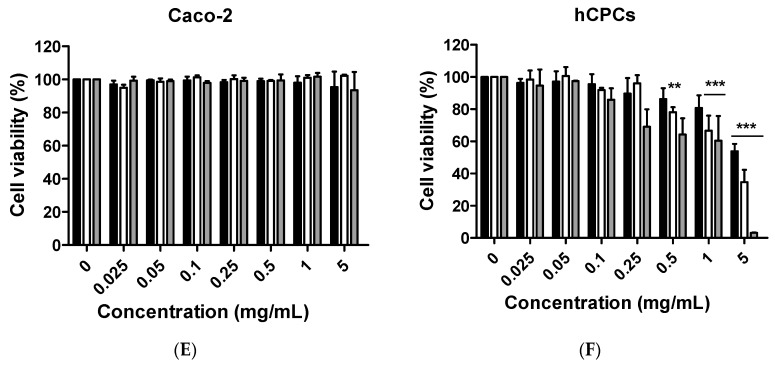
Cell viability measurement of citron, citron_peptide_, and citron_liposome_ by MTT assay. (**A**) HepG2 cells were cultured to the indicated time in 96-well plates. Then, the cells were treated with increasing concentrations of tested samples, as shown in the figure. After incubation for 24 h, cell viability was measured by MTT assay as described in “Materials and Methods”; (**B**) primary hEPC cell line; (**C**) NIH-3T3 cell line; (**D**) H9C2 cell line; (**E**) Caco-2 cell line; and, (**F**) primary hCPC cell line. The data were analyzed by using GraphPad Prism 5.0. Representative data is shown from three independent experiments with similar outcomes. Color in graph: Black, Citron; White, Citron_peptide_; Gray, Citron_liposome_. Significant results from the control group are calculated and marked with asterisks ** *p* < 0.01, *** *p* < 0.001).

Six different cell lines (HepG2, NIH-3T3, Caco-2, H9C2, primary hCPCs, and primary hEPCs) were employed to test the cell viability by the MTT method. As can be seen from Figure 2, neither citron, citron_peptide_ or citron_liposome_ induced any cell toxicity in the HepG2, NIH-3T3, and Caco-2 cell lines, even at the highest tested concentration of 5 mg/mL (Figure 2B,C,E). Interestingly, a dose-response pattern was apparently seen in the other three cell lines. In other words, the cell viability decreased with the increasing citron extract concentration. For instance, after treatment of citron and citron_peptide_, no effect was observed on H9C2 cells (Figure 2D), but in primary hCPCs and hEPCs, the percentage of living cells was reduced to around 50% at 5 mg/mL (Figure 2B,F). In addition, citron_liposome_ exhibited higher toxic effects than did citron and citron_peptide_ against H9C2 (Figure 2D), hCPCs, and hEPCs, with only 4% cell survival in hCPCs at 5 mg/mL (Figure 2F). It was clear that cytotoxicity of citron extracts showed cell type selectivity.

### 2.3. Visualization of Cell Viability

**Figure 3:**
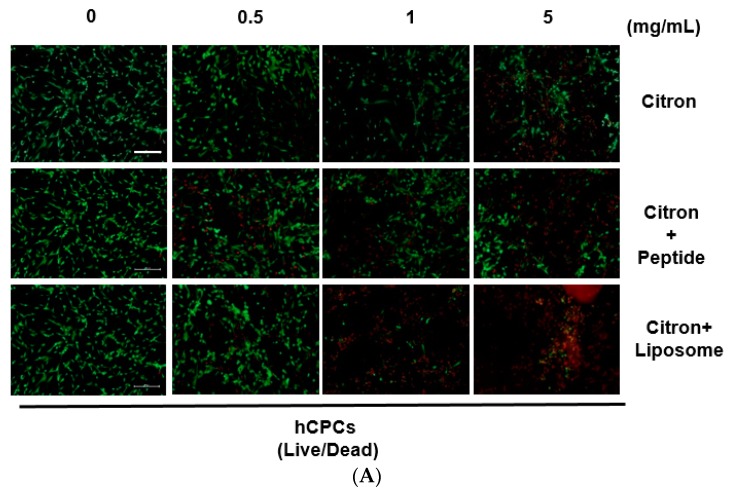

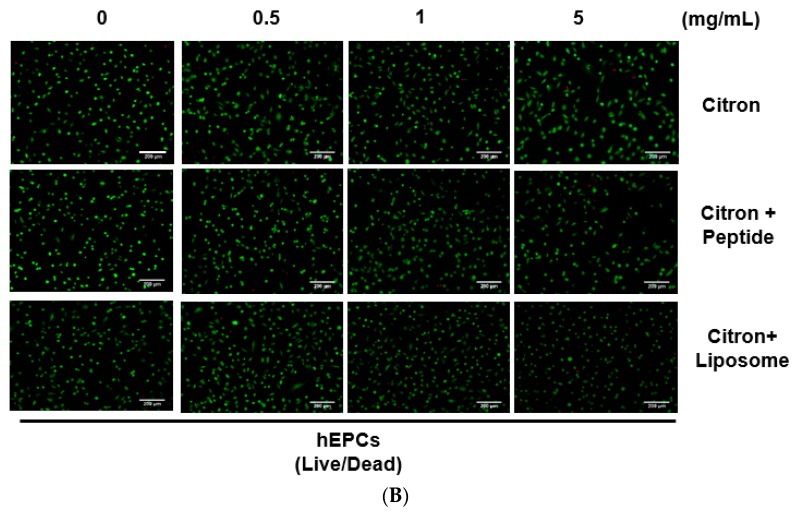
Visualization of Cell cytotoxicity by Live/Dead assay. (**A**) The primary hCPC cells were seeded into 48-well plates for this experiment. Following 24 h incubation after treatment of tested samples, the cells were stained by using fluorescent dyes according to the Live/Dead assay kit. The living cells are shown in green, the dead cells in red. The merged images in the figure show co-distribution of live and dead cells; (**B**) The primary hEPC cell line. Scale bar is 200 µm.

To further observe the cell cytotoxicity, the Live/Dead assay was conducted to visualize live and dead cells (shown in merged images in Figure 3 and Figure S1). The results were consistent with Figure 1. In Figure S1, no difference can be observed between the HepG2, NIH-3T3, Caco-2, and H9C2 cell lines (data only shows 5 mg/mL concentration). As expected, in Figure 3A, the ratio of live/dead cells increased in proportion to the sample concentration for hCPCs, but no difference could be distinguished in hEPCs (Figure 3B).

### 2.4. Measurement of Cytotoxicity

**Figure 4:**
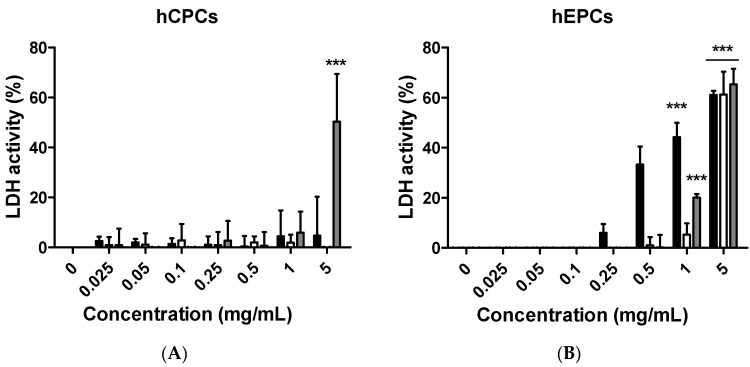

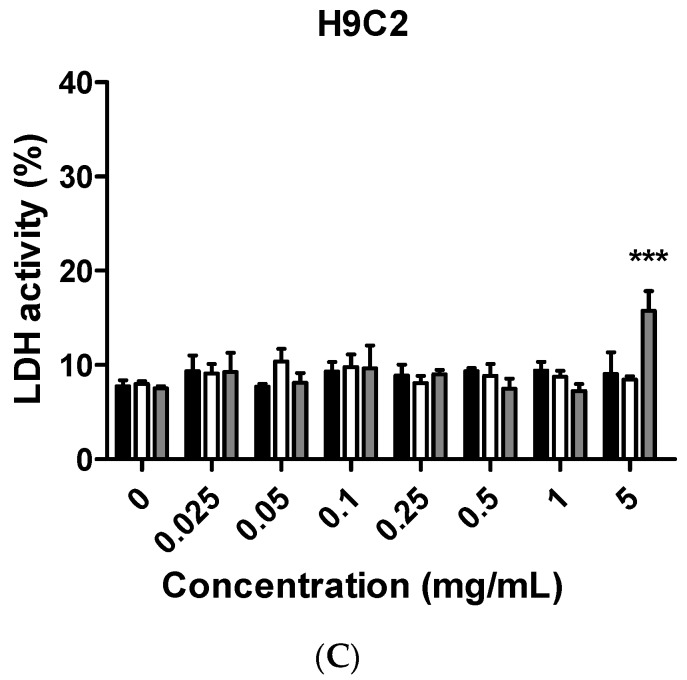
Cytotoxicity measurement by EZ-LDH assay. (**A**) hCPC cells were cultured in 96-well plates. Then, the cells were incubated with different concentrations of citron extracts. After incubation for 24 h, cell cytotoxicity was measured by using EZ-LDH assay kits, according to the manufacturer’s introduction; (**B**) primary hEPC cell line; (**C**) H9C2 cell line. GraphPad Prism 5.0 was used to analyze data and make graphs. The experiments were repeated three times with similar outcomes. Color in graph: Black, Citron; White, Citron_peptide_; Gray, Citron_liposome_. Significant results from the control group are calculated and marked with asterisks (*** *p* < 0.001).

Figure 4 shows the effects of citron extracts on the cytotoxicity, as measured by the lactate dehydrogenase (LDH) release assay. The cytotoxicity did not significantly differ from the lowest concentration to the highest concentration, including the control group, in HepG2 and NIH-3T3 cells (Figure S2). However, both primary hCPCs and hEPCs were affected by the addition of the citron samples and showed cell death up to 50% at 5 mg/mL treatment (Figure 4A,B). Consistent with the result in Figure 1D, citron_liposome_ showed a higher LDH activity than citron and citron_peptide_ in H9C2 cell line (Figure 4C).

### 2.5. Measurement of Genotoxicity

**Figure 5:**
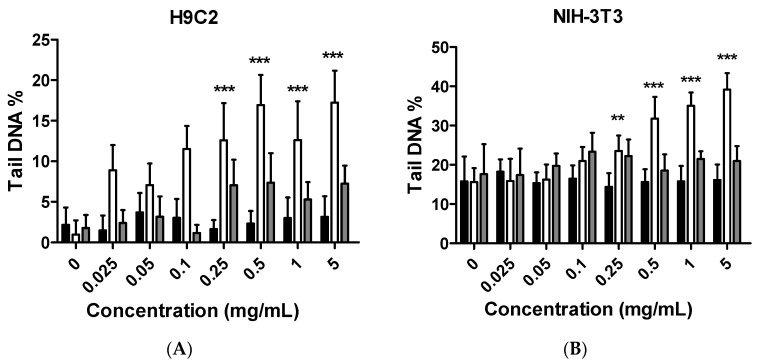

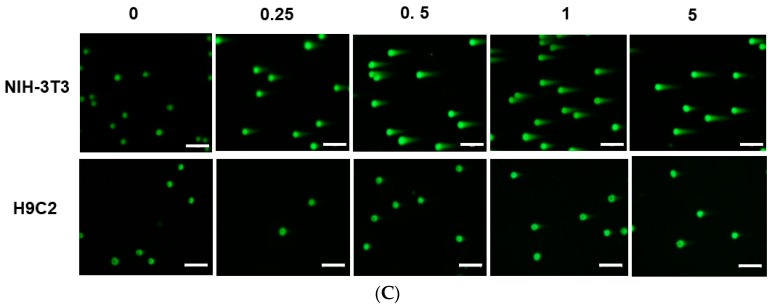
Genotoxicity measured by comet assay. (**A**) The H9C2 cells were seeded into 48-well plates to perform this experiment. Following 24 h incubation after treatment of tested samples, the assay was carried out according to the comet assay protocol as described in “Materials and Methods”; (**B**) NIH-3T3 cell line. Color in graph: Black, Citron; White, Citron_peptide_; Gray, Citron_liposome_. Significant results from the control group are calculated and marked with asterisks (** *p* < 0.01, *** *p* < 0.001); (**C**) The typical comet assay images in the Citron_peptide_ group are shown at the concentration of 0.25, 0.5, 1, 5 mg/mL. Scale bar is 100 μm.

To evaluate whether the citron extracts affect genes, a single-cell gel electrophoresis assay (comet assay) was performed with the hCPCs, H9C2, and NIH-3T3 cell lines. Surprisingly, although citron extracts can cause cell cytotoxicity in hCPCs, with over 90% mortality of cells in the citron_liposome_ group (Figure 2F), there was no detectable genotoxicity in the same cell line (Figure S3). In contrast, in the H9C2 and NIH-3T3 cells, as shown in Figure 5A,B, the proportion of tail DNA increased after citron_peptide_ treatment, but no genotoxicity was observed in either the citron group or the citron_liposome_ group. Interestingly, the genotoxicity disappeared in the presence of liposome in the citron_peptide_ extract. Figure 5C displays the toxicity of citron_peptide_ on genes in H9C2 and NIH-3T3 cells. It was evident that citron_peptide_ selectively caused gene damage in NIH-3T3 cells. With the liposome in the citron extract to form nanoparticles, the damage to genes can be suppressed or abolished (Figure 5A,B).

## 3. Discussion

Functional foods, as one area of the food industry, have grown a lot over the years [18]. Benefiting from the improvement of food technology, especially with the emergence of nanotechnology, it is expected that the functional foods will further advance, with promising prospects [19]. In this study, by using nanotechnology, we prepared citron extract at a nanometer scale by incorporating functional ingredients, liposome, and peptide. We employed fish oligopeptide and water-soluble liposome to formulate citron nanoparticles. With predominant properties in viscosity and foaming, oligopeptide has been widely used in food processing. Meanwhile, because of its high yield, low cost, and the beneficial effects in human body, we used fish collagen peptide to process citron_peptide_ into nanoparticle, as shown in Figure 1A. The typical size of citron_peptide_ nanoparticles was around 100 nm. However, because of the biological limitation of oligopeptide, such as chemical degradation, low stability, and bioavailability, physicochemical instability, and low permeability, its development in the food industry is restricted. To avoid the deficiencies of peptide, we used both liposome and oligopeptide to formulate citron nanoparticles, labeled as citron_liposome_ in Figure 1A. The content ratio of three types of citron samples (Citron, Citron_peptide_, and Citron_liposome_) are depicted in supplementary data Table S1. Furthermore, the description of the citron extract compositions such as general composition (Table S2), amino acid (Table S3), fatty acid (Table S4), minerals (Table S5), and vitamins (Table S6) are provided in supplementary data file as well (obtained from Rural Development Administration, Korea). 

Next, regarding the determination of citron nanoparticle, the morphology of citron_peptide_ and citron_liposome_ clearly demonstrated the formation of citron extracts nanoparticles. On the other hand, concerns remain with the significantly reduced size of particles when utilizing nanotechnology. For example, it has been reported that particles with a diameter of less than 100 nm may induce toxic effects [20]. To assess the toxicity of citron nanoparticles and provide quantitative references for various applications, we confirmed the biological toxicity, as shown in the results section. 

Although citron is well-known for its medicinal usage, we know little about its application as food and its bioactivities. Here, in an in vitro test, six different cells were used to measure the toxicity of citron extracts. Embryonic fibroblasts from mice (NIH-3T3), heart myoblasts from rats (H9C2), a human liver-cancer cell line (HepG2), and human epithelial colorectal adenocarcinoma cells (Caco-2) were selected to test the different effects. In addition, we also used two human-derived primary cells, human cardiac progenitor cells (hCPC) and human endothelial progenitor cells (hEPC), to examine the toxicity and make the results more credible. Our work showed that the cytotoxicity of citron extract had two aspects. One aspect was that the cytotoxicity was cell-type dependent (Figure 2), selectively against primary hCPCs and hEPCs, especially in the citron_liposome_ group. The other was the genotoxicity (Figure 5). No cytotoxicity was observed in NIH-3T3 and H9C2 cell lines, but citron_peptide_ can cause genotoxicity in those two cell lines. Interestingly, in the presence of liposome in citron_liposome_, the gene damage disappeared. With the particles being in the nanometer scale, nanoparticles may penetrate biological membranes and have access to the cell. Nanoparticle can also have different biodistributions in the human body, perhaps because of different affinities with the diverse cell types or organs. In recent years, some studies have explored the nanotoxicity of nanoparticles [21,22]. In this view, we can conclude that citron extract behaved differently for different cell types. For instance, citron_liposome_ may show a higher affinity with hCPCs and hEPCs than other cell lines to cause cell damage (Figure 2), and with high affinity for genes, citron_peptide_ can have a genotoxic effect in the H9C2 or NIH-3T3 cell lines (Figure 5). We also discovered that citron extracts did not suppress human liver cancer cell lines or human epithelial colorectal adenocarcinoma cells, HepG2 and Caco-2.

The main purpose of this study is to identify the potential toxicity of citron nanoparticles (citron_peptide_ and citron_liposome_) in an in vitro system, and to provide accessible information for further industrial production and application. Many studies have shown that nanotoxicity can emerge with the decreased particle size to nanometer scale. Because the size of citron_peptide_ and citron_liposome_ is between 100 and 200 nm, it is essential to examine their toxicity. Our results demonstrate that cytotoxicity increased in some cell lines, like hCPCs, hEPCs, and H9C2, after treatment with citron_peptide_ or citron_liposome_. Meanwhile, citron_peptide_ caused genotoxicity in the NIH-3T3 and H9C2 cell lines, but not cytotoxicity. These results clearly show the toxic effects of citron nanoparticles. In addition, although our studies provide information about the cytotoxicity of citron for applications in the food industry, more research is needed to reveal the mechanism before use in final food products.

## 4. Materials and Methods

### 4.1. Chemicals and Kits

Trizma base, *N*-laurylsarcosine, Triton X-100, boric acid, and 3-(4,5-Dimethylthiazol-2-yl)-2,5-diphenyltetrazolium bromide (MTT) were obtained from Sigma-Aldrich (Madison, WI, USA). The cell-culture medium and dish were acquired from Welgene (Daegu, Korea). Fetal bovine serum (FBS) was bought from Welgene (Daegu, Korea). The LIVE/DEAD^®^ Viability/Cytotoxicity Assay Kit was purchased from Invitrogen (Carlsbad, CA, USA). The lactate dehydrogenase (LDH) Cytotoxicity Assay kit was obtained from Thermo Fisher Scientific (Waltham, MA, USA). All of the other reagents and chemicals were purchased from commercial companies.

### 4.2. Preparation of Samples

The method used for preparation of liposome has been described previously. Briefly, the 2% lecithin (Metarin P, Cargill Texturizing Solutions, Hamburg, Germany) was added to 0.5% peptide (Naticol 4000, Weishardt International, Graulhel, France) aqueous solution at a ratio of 1:1. Then, after homogenization for 3 min at 8000 rpm using a high-speed homogenizer (IKAT25 digital ULTRA-TURRAX^®^, Germany), the extracts were collected and homogenized again at 80 W for 3 min by using an ultrasonicator. The citron extract containing 50% citron and 50% sucrose was prepared by mixing with water (1:1 *w*/*w*) using a blender to make citron syrup for further study. The citron_peptide_ was obtained by mixing with fish skin peptide. Sixty grams of peptide were used per 0.5 L of citron extract. The citron_liposome_ was obtained by mixing with fish-skin peptide and liposome. The ratio was 1 g liposome and 60 g fish-skin peptide per 0.5 L of citron extract. Finally, all of the samples were dried using a spray dryer (SD-1000, EYELA, Tokyo, Japan) to obtain a powder. The morphology and size of citron extracts were detected by using scanning electron microscope (SEM) and Nano Size analyzer.

### 4.3. Cell Culture

NIH-3T3 (mouse), H9C2 (rat), and HepG2 (human) were purchased from Korean Cell Line Bank (KCLB, Seoul, Korea) and cultured in high glucose Dulbecco’s Modified Eagle’s Medium (DMEM; Welgene, Daegu, Korea) containing 10% FBS and 1% penicillin/streptomycin (P/S, Welgene). Primary human EPCs and human CPCs were provided by S.M. Kwon (IRB No. 05-2-15-133, Pusan University, Pusan, Korea). The hEPCs were cultured in EC basal medium 2 (EBM-2MV, Lonza, Walkersville, MD, USA) containing 5% FBS, EGM-2-MV Bullet Kit, and 1% penicillin/streptomycin. The hCPCs were cultured in Ham’s F12 medium (Hyclone, Logan, UT, USA) containing 10% FBS, 1% penicillin/streptomycin, 0.005 U/mL human erythropoietin (hEPO, R & D system, Minneapolis, MN, USA), 5 ng/mL human basic fibroblast growth factor (hbFGF, PeproTech, Rocky Hill, NJ, USA), and 0.2 mM L-glutathione reduced (Sigma-Aldrich, St. Louis, MO, USA). All cell lines were cultured at 37 °C in a humidified incubator with 5% CO_2_ atmosphere. The logarithmic cells were harvested before performing each experiment.

### 4.4. MTT Assay

The cell viability was measured by employing an MTT assay as described previously [23]. The cells were seeded into 96-well plates at a density of 1 × 10^5^ cells per well and were cultured overnight. After incubation, the medium was replaced by a starvation medium containing 1% FBS for another 24 h. Then, the cells were treated with the desired concentrations of drugs for an additional 24 h. The supernatant was removed, and an MTT solution at a final concentration of 0.25 mg/mL was added in each well to label the cells. The plates were incubated in a humidified incubator for 4 h. Then, 100 μL of dimethyl sulfoxide (DMSO) was used to dissolve the formazan, and the cell viability was detected by measuring the optical density (OD) at the absorbance of 595 nm using BioTek Microplate Readers (Winooski, VT, USA).

### 4.5. Live/Dead Assay

To visualize the cell viability, the cells were labeled with calcein AM and ethidium homodimer (EthD-1) by using a Live/Dead assay kit. The cells were cultured in 48-well plates at a density of 1 × 10^5^ cells per well for 24 h. After incubation with a starvation medium overnight, the various concentrations of drugs were added in each well and were incubated for the next 24 h. Then, the supernatant was discarded, and the cells were washed once using DPBS. A 500 μL cell-staining solution at a final concentration of 2 µM calcein AM and 4 µM EthD-1 in DPBS was added to each well, and the plates were incubated for 45 min at room temperature by blocking light. Finally, live and dead cells were visualized by using a fluorescence microscope (Nikon, ECLIPSE Ts2, Tokyo, Japan). The live cells are shown in green, the dead cells in red. The images merged by Image J software were used to show co-localization.

### 4.6. LDH Release Assay

Cell cytotoxicity was evaluated by measuring the enzymatic activity of released LDH from dead cells. Before the measurement, the cells were prepared, as described previously, and then incubated in various concentrations of drugs for 24 h. The plates were then centrifuged at 700× *g* for 5 min at room temperature using a Beckman Coulter Allegra^®^ X-15R centrifuge. The supernatant was collected to measure the released LDH. The assay was performed according to the manufacturer’s instructions. The absorbance at 450 nm was detected to calculate the cell cytotoxicity.

### 4.7. Single-Cell Gel Electrophoresis Assay

A single-cell gel electrophoresis assay (comet assay) was carried out to measure the deoxyribonucleic acid (DNA) damage. The method used for this assay analysis has been described previously. Briefly, cells were first seeded into 48-well plates. After incubation with different concentrations of drugs for 24 h, the cells were harvested and mixed with 100 µL 0.7% low-melting-point agarose at a cell density of 2 × 10^5^ per milliliter. Then, the cells were laid onto agarose-coated glass slides, which were stored at 4 °C for solidification until electrophoresis. Slides were placed in a lysis solution (2.5 M NaCl, 0.1 M EDTA, 10 mM Trizma base, 1% *N*-laurylsarcosine, 0.5% Triton X-100, and 10% DMSO, pH 10) for 1 h at 4 °C. After lysis, slides were submerged in an alkaline solution (300 mM NaOH, 1 mM EDTA, pH > 13) for 30 min at 4 °C. The slides were washed three times with electrophoresis buffer (89 Mm Trisbase, 89 mM boric acid, 2 mM EDTA, pH 8.3) and then subjected to electrophoresis at 25 V for 20 min using a RunOne^TM^ Powder Supply. Then, slides were fixed with absolute ethanol and stained with DNA dye. The cell images were photographed using a fluorescence microscope and Casplab software was used to analysis the comet assay results.

### 4.8. Statistics

All data were analyzed by GraphPad Prism and shown as means ± standard deviation (SD). Comparisons between the groups were conducted by using two-way analysis of variance with Bonferroni post-tests and/or Student’s *t* test. Statistical significance was considered to be a *p* value less than 0.05.

## 5. Conclusions

In this study, toxicity studies were conducted to determine the possible toxic effects of nano-engineered citron samples in the cellular level and to provide credible information for the future application of citron. To make the result more convincing, two human primary cell lines were employed in our study. Our findings revealed that citron_liposome_ could significantly cause cell damage in primary hEPC and hCPC cells, much greater than the slight damage caused by citron or citron_peptide_. However, cytotoxicity was not observed from NIH-3T3, HepG2, and Caco-2 cell lines. In the genotoxicity test, significant toxic effects were not observed from hEPCs or hCPCs, followed by the treatment with citron, citron_peptide_, or citron_liposome_. Minimal genotoxicity was detected from NIH-3T3 cells and in H9C2 cells after the treatment with citron_peptide_, but disappeared in the presence of liposome in the citron_liposome_ group. Overall, our findings provide important information for the further use of citron in the food industry. Moreover, in vitro study will be performed to further determine the toxicity in order to meet the final food products.

## Supplementary Materials

Supplementary materials can be found at http://www.mdpi.com/1422-0067/19/2/626/s1.

## Abbreviations

hEPCs	Human endothelial progenitor cells
hCPCs	Human cardiac progenitor cells
LDH	Lactate dehydrogenase
FBS	Fetal bovine serum
P/S	Penicillin/Streptomycin
