# Supplementary material for: Identification and Evaluation of Cytotoxicity of Peptide Liposome Incorporated Citron Extracts in an in Vitro System
Source: Int J Mol Sci. 2018 Feb 22;19(2):626. doi: 10.3390/ijms19020626 (PMC5855848; doi:10.3390/ijms19020626)
Supplement: Supplementary file 1 [file ijms-19-00626-s001.pdf]

Supplementary Materials: Identification and Evaluation of Cytotoxicity of Peptide Liposome Incorporated Citron Extracts in an *in Vitro* System

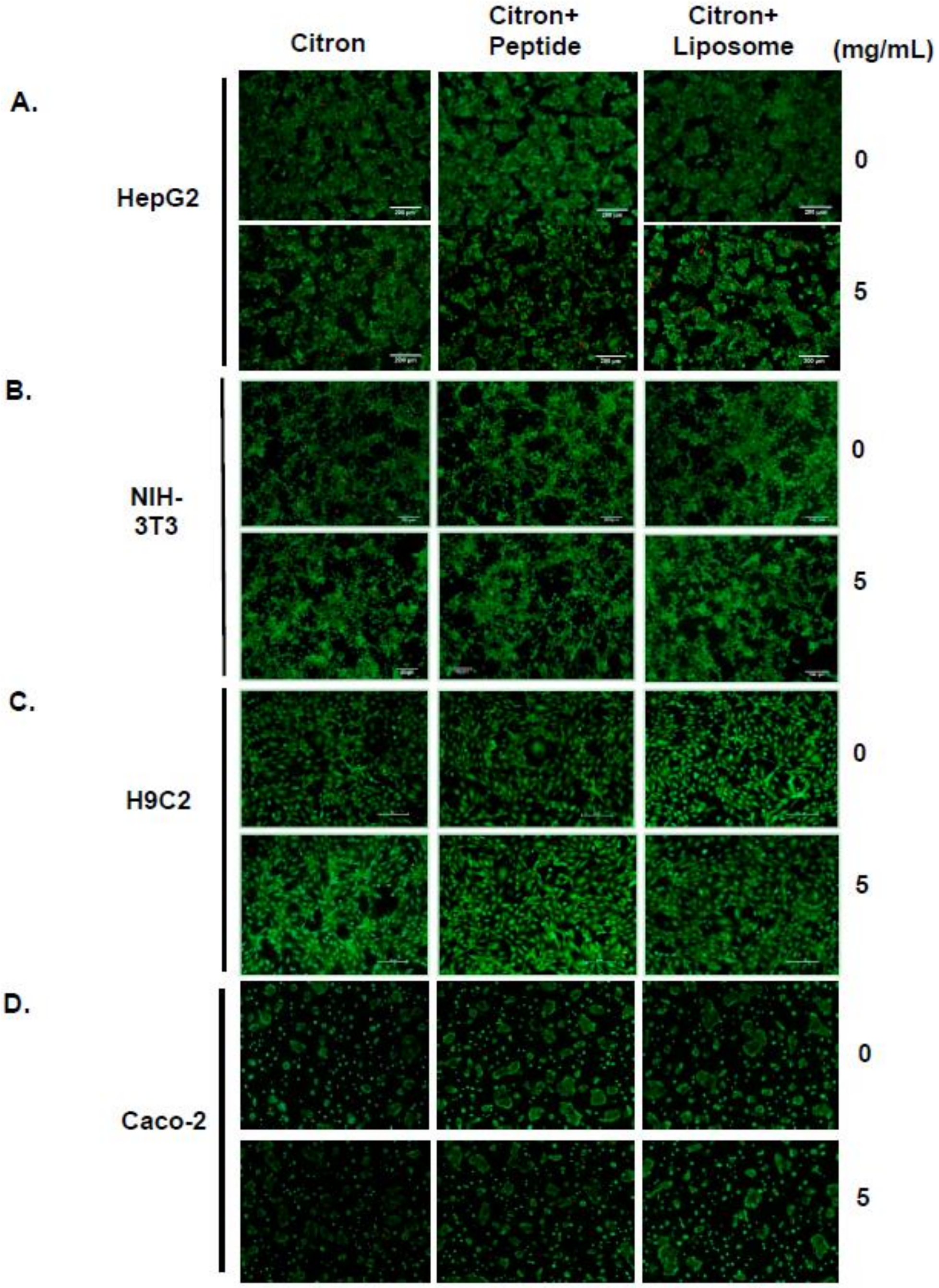

Figure S1. Visualization of Cell cytotoxicity by Live/Dead assay. (**A**) The HepG2 cells were seeded into 48-well plate. Followed by 24 h incubation after the treatment of tested samples, the cells were stained by using fluorescent dyes according to Live/Dead assay kit. The merged images showed in figure were to show co-distribution of live and dead cells; (**B**) NIH-3T3 cells; (**C**) H9C2 cells; (**D**) Caco-2 cells.

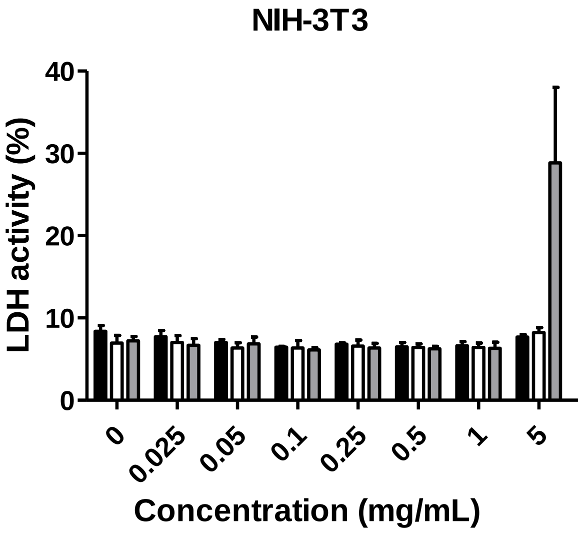

(**A**)

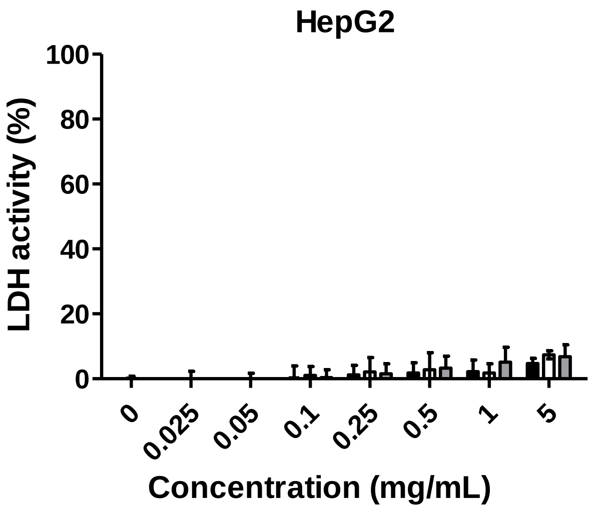

(**B**)

Figure S2. Cytotoxicity measurement by EZ-LDH assay. (**A**) NIH-3T3 cells were cultured in 96-well plate. Then, the cells were incubated with different concentration of citron extracts. After incubation for 24 h, cell cytotoxicity was measured by using EZ-LDH assay kits according to the manufacturer's introduction; (**B**) HepG2 cells. The program GraphPad Prism 5.0 was performed to analysis data and make graphs. The experiments were repeated three times with similar outcomes. Color in graph: Black-Citron; White-Citron$_{peptide}$; Gray-Citron$_{liposome}$.

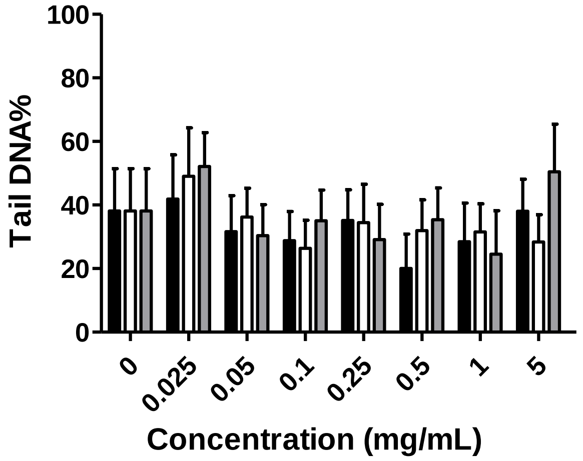

Figure S3. Genotoxicity determination by comet assay. The hCPCs were seeded into 48-well plate to perform this experiment. Following 24 h incubation after treatment of tested samples, the assay was carried out according to the comet assay protocol as described in "Materials and Methods". Color in graph: Black-Citron; White-Citron$_{peptide}$; Gray-Citron$_{liposome}$. Scale bar is 100 µm.

Table S1. Content ratio of citron samples (Citron, Citron $_{peptide}$, and Citron $_{liposome}$).

Sample	Citron extract (g)	Peptide (g)	Lecithin (g)	DW (mL)
Citron	25	-	-	50
Citron $_{peptide}$	25	3	-	75
Citron $_{liposome}$	25	3	0.5	75

DW: distilled water

Table S2. General composition of citron extract.

Ingredient	Content (g)
Moisture	86
Protein	0.93
Lipid	0.09
Ash	0.44
Carbohydrate	12.54
Sugars	4.1
Sietary fiber	8.2

The data were calculated by using 100 g citron.

* Data obtained from Rural Development Administration, Korea.

Table S3. Components of total amino acids of citron extract.

components	Content (mg)
Total amino acids	909
Essential amino acids	239
Non-essential amino acids	670

The data were calculated by using 100 g citron.

* Data obtained from Rural Development Administration, Korea

Table S4. Components of fatty acid of citron extract.

Fatty acid	Content (g)
Total fatty acid	0.08
Essential fatty acid	0.05
Saturates fatty acid	0.02
Monounsaturated fatty acid	0.01
Polyunsaturated fatty acid	0.05

The data were calculated by using 100 g citron.

* Data obtained from Rural Development Administration, Korea

Table S5. Components of minerals of citron extract.

Minerals	Content (mg)
Ca	36
Fe	0.16
Mg	12
P	15
K	17
Na	3
Zn	0.39
Mn	0.13
Mo	2.2×10^{-4}

The data were calculated by using 100 g citron.

* Data obtained from Rural Development Administration, Korea.

Table S6. Components of vitamins of citron extract.

Vitamins	Content (mg)
Beta-carotene	$8x10^{-3}$
Vitamin E	1.22
Vitamin B1	0.15
Vitamin B2	0.05
Niacin	0.1
Vitamin B5	1.62
Biotin	$2.79x10^{-3}$
Folic acid (DFE)	$1.5x10^{-2}$
Vitamin C	95.03

The data were calculated by using 100 g citron.

* Data obtained from Rural Development Administration, Korea.
